# Expression of angiogenic factors predicts response to chemoradiotherapy and prognosis of oesophageal squamous cell carcinoma

**DOI:** 10.1038/sj.bjc.6600129

**Published:** 2002-02-12

**Authors:** H Shimada, T Hoshino, S Okazumi, H Matsubara, Y Funami, Y Nabeya, H Hayashi, A Takeda, T Shiratori, T Uno, H Ito, T Ochiai

**Affiliations:** Department of Academic Surgery, Chiba University Graduate School of Medicine, 1-8-1 Inohana, Chuou-ku, Chiba 260-8677, Japan; Department Radiology, Chiba University Graduate School of Medicine, 1-8-1 Inohana, Chuou-ku, Chiba 260-8677, Japan

**Keywords:** oesophageal cancer, chemoradiotherapy, thymidine phosphorylase, vascular endothelial growth factor, treatment response

## Abstract

The ability to predict patients' responses to chemoradiotherapy by analyzing pre-treatment biopsy specimens would be valuable for managing oesophageal squamous-cell cancer. To this end, the expression of p53, thymidine phosphorylase and vascular endothelial cell growth factor was analyzed by immunohistochemistry in 52 patients with oesophageal squamous-cell cancer prior to chemoradiotherapy. Treatment consisted of radiotherapy (40 Gy) and 5 day-infusion of 5-Fluorouracil (500 mg m^−2^ per day) combined with cisplatin (10 mg m^−2^ per day). Following treatment, imaging and endoscopic reassessment was performed to establish treatment response. Thirty-one patients underwent radical surgery and 21 patients were treated with an additional 20 Gy of radiotherapy. Of the tumours studied, 58% were p53-positive, 40% thymidine phosphorylase-positive and 44% vascular endothelial cell growth factor-positive. A clinical response was observed in 36 patients (69%) and was negatively associated with thymidine phosphorylase expression (*P*=0.02) and vascular endothelial cell growth factor expression (*P*<0.001). However, the 5-year survival rate was significantly lower only in patients with vascular endothelial cell growth factor-positive tumours (*P*=0.037). Multivariate analysis identified vascular endothelial cell growth factor as a significant independent prognostic factor (*P*=0.0147). These results suggest that expression of angiogenic factors has predictive value for the treatment response and outcome of patients with oesophageal cancer.

*British Journal of Cancer* (2002) **86**, 552–557. DOI: 10.1038/sj/bjc/6600129 www.bjcancer.com

© 2002 Cancer Research UK

## 

Despite improvements in surgical techniques, rapid fatal recurrence is common in patients with advanced oesophageal cancer (Isono *et al*, 1982, 1990). Because surgical resection alone rarely results in long-term survival, efforts are now focused on combined multi-modality treatments in an attempt to improve local control and eliminate micro-metastasis present at the time of surgery.

Recently, although neo-adjuvant chemoradiotherapy (CRT) followed by oesophagectomy has become widespread after several favourable pilot studies were reported (Poplin *et al*, 1996; Stahl *et al*, 1996; Ancona *et al*, 1997; Ide, 1997), contradictory data have also been published (Bosset *et al*, 1997; Tamin *et al*, 1998). A major problem in this context seems to be that a lower rate of cancer-related deaths after combined treatment is counterbalanced by a higher rate of treatment-associated mortality. Because only patients with potentially responsive tumours would benefit from such aggressive treatment, prediction of treatment response by means of tissue analysis is invaluable in the management of these patients with advanced oesophageal cancer. If non-responsive tumours could be identified, these patients could be spared the significant toxicity, time, and financial expense associated with intensive therapeutic regimens. Determination of appropriate pre-treatment factors necessary for prediction of patients' responses to CRT is vital and can be achieved by ‘biological staging’ using predictive biological factors. Therefore, a primary consideration in setting up CRT for patients with advanced oesophageal cancer is to identify markers serving as good predictors for treatment response.

Given the importance of alterations in the p53 gene and expression of angiogenic factors for progression of oesophageal cancer, it is reasonable to explore whether such markers may have predictive value for the patients' response to therapy. Indeed, genetic alteration of p53 or p53 protein over-expression has already been reported to be a good predictor for treatment response and survival in oesophageal cancer (Sarbia *et al*, 1994; Nabeya *et al*, 1995; Casson *et al*, 1998; Ribeiro *et al*, 1998; Yang *et al*, 1999; Kobayashi *et al*, 1999; Shimada *et al*, 2000).

Angiogenesis plays an essential role in the process of growth and metastasis of solid tumours (Weidner, 1995, Hanahan and Folkman, 1996). Among several angiogenic factors, vascular endothelial cell growth factor (VEGF) has been shown to be vital for pathological angiogenesis. VEGF induction and vascularization of solid tumours has been shown to play an important role in the response to chemotherapeutic agents and radiation therapy (Shintani *et al*, 2000; Veikkola *et al*, 2000; Volm and Rittgen, 2000). Immunohistochemical (IHC) analyses of oesophageal carcinoma have revealed that angiogenesis, as determined by micro-vessel density, is a prognostic factor (Inoue *et al*, 1997; Kitadai *et al*, 1998; Sato *et al*, 1999; Shih *et al*, 2000). Over-expression of VEGF protein is, therefore, at least partially responsible for the malignant potential in oesophageal cancer and represents a useful prognostic marker. Thymidine phosphorylase (TP, EC 2.4.2.4), which is identical to platelet-derived endothelial cell growth factor, is also a potent angiogenic factor (Griffiths and Stratford, 1997). In oesophageal squamous-cell carcinoma (SCC), IHC studies indicated that high TP expression was associated with angiogenesis, tumour progression and poor prognosis (Igarashi *et al*, 1998; Takebayashi *et al*, 1999). In head and neck SCC, a low percentage of cancer cells with nuclear TP expression in pre-treatment biopsies was associated with a high rate of complete regression after combined CRT (Koukourakis *et al*, 2000). Although angiogenic factors were reported as prognostic indicators in oesophageal cancer after surgery, little information is available on their predictive value for the treatment response and their prognostic significance in patients receiving CRT.

In this report, we analyzed pre-treatment biopsy samples from 52 patients with primary oesophageal SCC by IHC to identify p53, TP and VEGF expression. We found that TP and VEGF expression were significantly associated with clinical responses to treatment. We also found that VEGF expression was an independent prognostic factor for patients with oesophageal SCC following CRT.

## MATERIALS AND METHODS

### Patients and samples

For inclusion in this study, patients were required to have presented at the Department of Academic Surgery, Chiba University Hospital, between 1991 and 1999 with histologically-proven primary SCC of the oesophagus and to have been treated by external beam radiotherapy concurrent with chemotherapy. A review of the clinical records identified 61 patients satisfying entry criteria. Nine of these were subsequently excluded because of the small size of the biopsy (five patients), previous or synchronous malignancies (two patients) or the existence of distant metastasis at the onset of treatment (two patients). The other 52 patients with primary advanced oesophageal squamous cell carcinoma underwent a prospective, non-randomized trial of combination CRT. The patients consisted of 43 males (83%) and nine females (17%), with a mean age of 65±9.6 years. Pre-treatment evaluation included clinical staging according to the TNM classification (Sobin and Wittekind, 1997), determined by radiography, endoscopic ultrasonography and computed tomography examinations ( Table 1

**Table 1:**
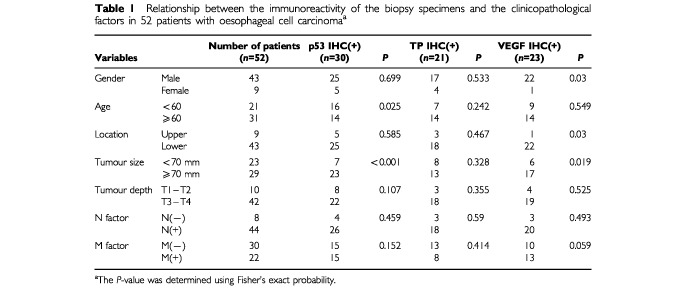
Relationship between the immunoreactivity of the biopsy specimens and the clinicopathological factors in 52 patients with oesophageal cell carcinoma^a^

). This study was reviewed and approved by the Chiba University School of Medicine Internal Review Board. Patient eligibility criteria included the following: (i) histologically confirmed SCC of the cervical and thoracic oesophagus; (ii) age limit of 80 years and Karnofsky performance status of greater than 70%; (iii) white blood cell count greater than 4000 cells mm^−3^, haemoglobin greater than 10 g, platelet count greater than 100 000 mm^−3^, creatinine less than 1.5 mg dl^−1^ and creatinine clearance greater than 50 ml min^−1^, total bilirubin less than 1.5 mg dl^−1^; and (iv) informed consent according to the Declaration of Helsinki present. At least three biopsy samples taken from different areas of the tumour of these patients for IHC analysis were obtained before treatment and stored until assay.

### Treatment plan

The chemotherapy schedule consisted of cisplatin 10 mg m^−2^ day^−1^ intravenous administration and 5-fluorouracil 500 mg m^−2^ day^−1^ in continuous intravenous infusion for 5 days. The radiotherapy dose of 2 Gy per day was initiated on day 1 of chemotherapy and continued daily for 5 days per week for 4 weeks, totalling 40 Gy. The target was the entire oesophagus as well as the supraclavicular lymph nodes for the upper and mid-third lesions. Coverage of the celiac lymph nodes was decided according to computed tomography examination. Of the 52 patients, 21 were treated with an additional 20 Gy of radiotherapy (to be a definitive CRT) and 31 were treated by transthoracic oesophagectomy. Postoperative treatment was not given. Resection of the oesophagus and the proximal stomach was performed by a combined right thoracic abdominal and cervical approach. Resection included excision of the para-oesophageal, paracardial, left gastric and celiac lymph nodes.

### Definition of the response to treatment

Re-evaluation of the primary tumour was performed by computed tomography, endoscopy and gastrography 2 weeks after completion of CRT. The response to treatment was basically evaluated according to the General Rules for Esophageal Cancer proposed by the Japanese Society for Esophageal Disease (1998) and was categorized as either a complete or partial response, stable or progressive disease. This evaluation was based on a comparison of initial and pre-operative imaging studies. A complete response was defined as the disappearance of all signs and symptoms of the tumour. A partial response was defined as a reduction of 50% or more of the tumour volume and microscopic evidence of residual tumour in postoperative specimens. The sum of the perpendicular diameter of the lesion was used to calculate tumour volume. Stable disease was defined as less than a 50% decrease or less than a 25% increase in tumour volume. Progressive disease was defined as no significant change in tumour mass or more than a 25% increase in tumour volume. The patients who showed a response, complete or partial, were categorized as responders. The remaining patients with either stable or progressive disease, were categorized as non-responders.

All patients underwent clinical examination and imaging every 3 months for the first year after the end of treatment; thereafter, every 6 months. Thirty-four patients (65%) were followed until their deaths with a median follow-up period for survivors of 36 months.

### Immunohistochemical staining for p53, TP and VEGF

Paraffin-embedded tissue blocks of formalin-fixed three biopsy specimens from different areas of the tumour were processed for conventional histological assessment by hematoxylin and eosin (H&E) staining and IHC analysis by the avidin–biotin–peroxidase method (Hsu *et al*, 1981). p53, TP and VEGF protein over-expression in the biopsy specimens was detected by anti-p53 monoclonal antibody (DO-7, DAKO, Carpenteria, CA, USA), anti-human TP (Nippon Roche Research Center, Kamakura, Japan; Nishida *et al*, 1996) and anti-human VEGF (A-20, Santa Cruz Biotechnology, Inc., Santa Cruz, CA, USA) using conventional peroxidase methods (Ribeiro *et al*, 1998). In brief, 4 μm thick sections were deparaffinized in xylene, dehydrated through graded alcohol concentrations and incubated in citrate buffer (pH=6.0) for 5 min using a household microwave oven at 800 W. After microwave exposure, the slides were allowed to cool to room temperature. The slides were briefly washed with PBS and incubated for 15 min with 3% hydrogen peroxide in methanol to block endogenous peroxidase activity. The antibodies to p53, TP and VEGF were diluted 1 : 250, 1 : 500 and 1 : 100, respectively and incubated for 24 h at 4°C. Biotinylated anti-mouse/rabbit antibody (DAKO) at a dilution of 1 : 500 was used as the second antibody. After washing, ABC (DAKO) was applied and diaminobenzydine was used for visualization.

The stained sections were evaluated at a high magnification (×400). Staining was considered positive for p53 when more than 10% of the cells' nuclei were strongly stained. Staining was considered positive for TP or VEGF when more than 10% of the tumour cells were strongly stained. Evaluation of this immunoreactivity of three biopsy specimens was performed without knowledge of the patients' clinicopathological factors by two investigators simultaneously (T Hoshino and A Takeda). When more than two of three biopsy specimens revealed positive immunoreactivity, staining was considered positive.

### Statistical analyses

Fisher's exact probability test was applied to determine the significance of the difference between two groups. Actual 5 year survival rates were compared between the two groups. Survival probabilities were calculated by the product limit method of Kaplan and Meier. Differences between groups were tested using the log-rank test. The influence of each clinicopathologic variable on survival was assessed by Cox's proportional hazards model. All statistical analyses were carried out using the Stat View program (SAS Institute Inc., Cary, NC, USA), and all *P* values were considered to be statistically significant if <0.05.

## RESULTS

### Immunoreactivity and clinicopathological variables

**Figure 1:**
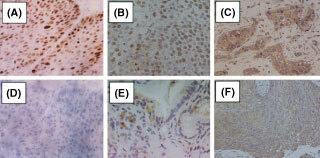
Representative p53, TP and VEGF immunoreactivity. (**A**) p53-IHC positive, (**B**) p53-IHC negative, (**C**) TP-IHC positive, (**D**) TP-IHC negative, (**E**) VEGF-IHC positive, (**F**) VEGF-IHC negative.

p53 expression was detected on the cells' nuclei. TP expression was detected on the cell cytoplasms, on the nuclei and on the some tumour-infiltrating stromal cells. VEGF expression was mainly detected on the cell cytoplasms or the membranes of the carcinoma cells ( Figure 1

). The overall frequency of expression of p53, TP and VEGF, summarized in Table 1, was 58% (30 of 52), 40% (21 of 52) and 44% (23 of 52), respectively. By p53-IHC, significant differences between positive and negative groups were observed for the factors age (*P*=0.025) and tumour size (*P*<0.001). By TP-IHC, no significant differences between the two groups were found. By VEGF-IHC, significant differences between the two groups were observed in terms of gender (*P*=0.03) and tumour size (*P*=0.019).

### Response to treatment and prognosis

**Figure 2:**
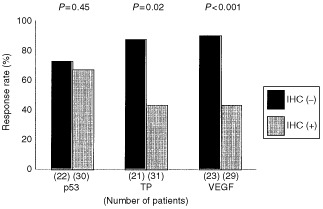
Relationship between immunoreactivity of the biopsy specimens and clinical responses to CRT in patients with oesophageal SCC. The *P*-values were determined by Fisher's exact probability testing.

**Figure 3:**
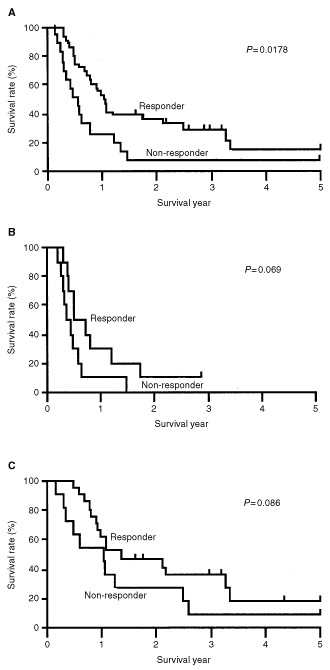
Kaplan–Meier survival according to treatment response and treatment modality. (**A**) All patients (*n*=52). (**B**) The patients having CRT alone (*n*=21). (**C**) The patients having neoadjuvant CRT followed by surgery (*n*=31). The *P*-values were determined using log-rank test.

Overall responses, including complete and partial responses, were observed in 36 patients (69%), with no response in the remaining 16 (31%). No significant differences were observed between the response rates of p53-ICH-positive or negative tumours (67 *vs* 73%, *P*=0.45) ( Figure 2

). However, the response rate of TP-ICH-positive tumours was significantly lower than TP-ICH-negative tumours (43 *vs* 87%, *P*=0.02). A similar tendency was observed for VEGF-ICH-positive or negative tumours (43 *vs* 90%, *P*<0.001). The overall 5-year survival rate was significantly higher in the responder group than the non-responder group (14.5 *vs* 6.3%, *P*=0.0178) ( Figure 3a

). A similar tendency was observed in each group of the patients having CRT only (Figure 3b) and the patients having CRT followed by surgery (Figure 3c). However, because the number of the patients in each group were not enough to reach statistically significant levels (*P*=0.069 and 0.089, respectively).

### Prognostic relevance and multivariate analysis

Using univariate analysis, treatment modality, tumour depth, N factor and VEGF-IHC status yielded a significant estimate of prognosis ( Table 2

**Table 2:**
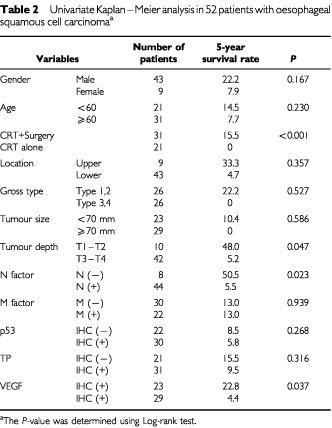
Univariate Kaplan–Meier analysis in 52 patients with oesophageal squamous cell carcinoma^a^

). In contrast, neither p53-IHC status nor TP-IHC status was informative for the prognosis after CRT in these oesophageal SCC patients. To determine independent prognostic values for patients' survival, a Cox's regression model was constructed using TNM factors and IHC status ( Table 3

**Table 3:**
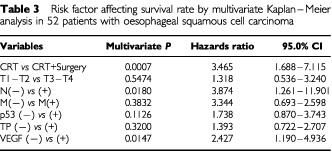
Risk factor affecting survival rate by multivariate Kaplan–Meier analysis in 52 patients with oesophageal squamous cell carcinoma

). VEGF-IHC was thereby identified as an independent predictor of patient survival (*P*=0.0147; hazards ratio, 2.424; 95% CI, 1.190–4.936). The other independent prognostic factors were treatment modality (P=0.0007; hazards ratio, 3.465; 95% CI, 1.688–7.115) and N factor (*P*=0.018; hazards ratio, 3.874; 95% CI, 1.261–11.901).

## DISCUSSION

In this study, the clinical significance of ICH-positivity for p53, TP and VEGF in pre-treatment biopsy specimens was examined in 52 patients with oesophageal SCC prior to CRT. Our results indicated that both TP and VEGF but not p53 expression was associated with treatment response. VEGF expression was also identified as an independent prognostic factor.

In contrast to previous reports (Ribeiro *et al*, 1998; Yang *et al*, 1999), we found that p53 expression was not a predictive indicator of treatment response. We suggest three possible explanations for p53-IHC status not being associated with either treatment response or survival. First, p53-ICH-negative cells also include instances of loss of both p53 alleles or nonsense mutations. Second, there may be a discrepancy in sequence analysis between assessments from different locations, i.e. endoscopic biopsy samples and surgically resected specimens. Endoscopic biopsy samples do not accurately represent characteristics of all tumour cells. In our other series, we compared the p53-IHC and TP-IHC staining results from the biopsy and resected specimens. The sensitivity was more than 90% and the specificity was around 80% (unpublished data). In this present study, because all patients received CRT, we could not compare the IHC staining results from the biopsy and resected specimens for the validity of the data. Third, there are significant differences in histology between the tumours examined in the previous reports and our study, because all of our cases were histologically proven to be SCC, whereas more than two-thirds of the previously-reported cases were adenocarcinomas. There might well be differences between the response rates of SCC and adenocarcinoma even with the same p53 mutations.

The association of angiogenic factor expression with a high incidence of treatment failure may contribute to the resistance to therapy observed in both the TP-ICH- and VEGF-ICH-positive groups. The duration to treatment failure and the treatment response rates were significantly poorer in the VEGF-ICH-positive group compared to the VEGF-IHC-negative group, and thus would eventually lead to poorer survival, as reported previously (Kitadai *et al*, 1998; Sato *et al*, 1999; Shih *et al*, 2000). Blocking VEGF activity was reported to enhance the anti-tumour effects of ionizing radiation (Gorski *et al*, 1999). Those authors proposed a model in which induction of VEGF by ionizing radiation contributes to the protection of tumour vessels from radiation-mediated cytotoxicity. In our present study, both the TP-ICH- and VEGF-ICH-positive groups experienced significantly lower treatment response rates. Therefore we propose a new model in which VEGF and TP expression both contribute to the protection of tumour blood vessels from CRT-mediated cytotoxicity and thereby to treatment resistance.

One question raised by the present study is why TP expression was not a significant prognostic factor, despite the fact that it was significantly associated with response rate. We suggest two possible reasons for this result. First, TP expression in our series was not associated with TNM factors, in contrast to previous reports (Igarashi *et al*, 1998; Takebayashi *et al*, 1999). Second, the complete response rate of TP-ICH-negative patients was relatively low compared to VEGF-ICH-negative patients. In the multivariate analysis, because TP expression was significantly associated with VEGF expression (*P*=0.03, data not shown), consistent with previous reports (Fujimoto *et al*, 1998; O'Byrne *et al*, 2000), VEGF might be identified as an independent prognostic factor in place of TP. As the small number of patients enrolled in this study was a limitation, further larger scale studies are required to address this question.

The development of convenient and reliable biomarkers predicting which patients are most likely to develop recurrence of primary disease would allow intervention strategies to be specifically targeted to patients most likely to benefit from them. Such a capability would be cost-effective and would avoid treating patients with a low response potential, who do not react to the usual adjuvant therapy. The present study suggests that patients with locoregional advanced oesophageal SCC positive for angiogenic factors are less likely to benefit from neoadjuvant CRT with the usual regimen than patients who are negative for angiogenic factors. Among 52 patients, 13 had tumours both TP- and VEGF-IHC-positive and a further 21 both TP- and VEGF-IHC-negative. Only three patients in the former group of 13 responded to therapy whereas 20 patients of the latter group of 21 did respond (data not shown). Moreover, none of the 13 in the TP- and VEGF-IHC-positive group survived >5 years. Therefore, monitoring angiogenic factors may be an important determinant for the differential application of therapy, not only for primary tumours but also for adjuvant therapy after definitive treatment of oesophageal cancer. It is essential to evaluate the prognosis separately in patients having neoadjuvant therapy with resection (*n*=31) and neoadjuvant therapy only (*n*=21) groups to confirm the prognostic value of VEGF-IHC. However, because a limited number of the patients in each groups, TNM factors and VEGF-IHC were assessed by multivariate analysis with treatment modality. Although neither p53-IHC nor TP-IHC were not independent prognostic factor, both VEGF-IHC and the treatment modality were selected as independent prognostic factors.

It is very difficult to develop an alternative treatment strategy for patients with tumours expressing angiogenic factors; however, radical surgery should at least be conducted without delay in these cases. VEGF-ICH-positive patients are deemed to have higher risks for recurrence and thus need more aggressive adjuvant therapy than the VEGF-ICH-negative group. Anti-VEGF therapy using anti-VEGF antibodies (Gorski *et al*, 1999; Lee *et al*, 2000) or anti-VEGF receptor therapy (Klement *et al*, 2000; Geng *et al*, 2001) may be useful in improving the effect of CRT and the prognosis of such VEGF-positive patients. Inhibitors of TP and prodrugs that are activated by TP (Ishikawa *et al*, 1998; Miwa *et al*, 1998; Takebayashi *et al*, 1999) may suppress the growth of TP-expressing tumours and may enhance the effect of CRT for patients with oesophageal SCC.

In conclusion, the present study indicates that monitoring the expression of angiogenic factors in biopsy specimens from patients with oesophageal SCC prior to treatment may have predictive value for their response to CRT and hence overall prognosis.
